# Mechanisms underlying delirium in patients with critical illness

**DOI:** 10.3389/fnagi.2024.1446523

**Published:** 2024-09-26

**Authors:** Ying-Ying Fan, Ruo-Yu Luo, Meng-Tian Wang, Chao-Yun Yuan, Yuan-Yuan Sun, Ji-Yong Jing

**Affiliations:** ^1^School of Nursing, Zhejiang Chinese Medical University, Hangzhou, China; ^2^Zhejiang Provincial People’s Hospital, People’s Hospital of Hangzhou Medical College, Hangzhou, China

**Keywords:** delirium, critical illness, cognition impairment, mechanism, review

## Abstract

Delirium is an acute, global cognitive disorder syndrome, also known as acute brain syndrome, characterized by disturbance of attention and awareness and fluctuation of symptoms. Its incidence is high among critically ill patients. Once patients develop delirium, it increases the risk of unplanned extubation, prolongs hospital stay, increases the risk of nosocomial infection, post-intensive care syndrome-cognitive impairment, and even death. Therefore, it is of great importance to understand how delirium occurs and to reduce the incidence of delirium in critically ill patients. This paper reviews the potential pathophysiological mechanisms of delirium in critically ill patients, with the aim of better understanding its pathophysiological processes, guiding the formulation of effective prevention and treatment strategies, providing a basis for clinical medication.

## 1 Introduction

In the Diagnostic and Statistical Manual of Mental Disorders, Fifth Edition, Text Revision (DSM-5-TR) (American Psychiatric Association, 2022; First et al., 2023), published by the American Psychiatric Association (APA), delirium is characterized by a disturbance in attention and additional disturbance in cognition that develop rapidly and exhibit fluctuations, often accompanied by reduced awareness of the environment. The disturbances is a direct physiological consequence of another medical condition, substance intoxication or withdrawal, or exposure to a toxin, or is due to multiple etiologies. The incidence of delirium varies across different settings, and for critically ill adult patients in the ICU, the occurrence rate of delirium is at least 30% (Brennan et al., 2023). Delirium is a clinical syndrome caused by a diversity of causes that might converge into a final pathophysiological pathway. It can manifest as different and overlapping phenotypes, primarily classified into three subtypes: hyperactive, hypoactive, and mixed. Among them, the hypoactive subtype is the most common in the ICU (Krewulak et al., 2018). However, it is often difficult to identify because its features include reduced motor activity, drowsiness, and diminished responsiveness. Patients with delirium have an increased risk of mortality during hospitalization. Moreover, the longer the duration of delirium, the poorer the overall cognitive and executive function after discharge. A 2-year follow-up study revealed that delirium patients had significantly lower physical functioning compared to non-delirium patients (Pandharipande et al., 2013; Lobo-Valbuena et al., 2023). This has a profound impact on the psychological well-being and quality of life of both patients and their families. Currently, there is no consensus regarding the mechanisms underlying delirium, nor is there agreement on optimal strategies for drug prevention and treatment. The incidence of delirium in the ICU remains high. The complex and not fully understood nature of delirium complicates the development of effective pharmacological interventions. Researchers face significant challenges in identifying effective drug targets, designing optimal experimental protocols, establishing subject selection criteria, and determining appropriate endpoint indicators. Additionally, predicting potential drug side effects adds to the complexity and risk of drug development. The lack of institutional clarity further complicates the formulation of clinical guidelines, leading to variability in treatment approaches among clinicians and potentially affecting the efficacy of clinical practice. Therefore, it is of great significance to delve into the pathophysiological mechanisms of delirium in order to timely control disease progression and explore potential pharmacological targets for prevention. This will help reduce the adverse effects on patients, improve the utilization of healthcare resources, and ultimately improve patient outcomes. The purpose of this study is to provide a comprehensive review of the latest research on the pathogenesis of ICU delirium and to explore potential future research directions.

## 2 Association between ICU delirium and patient characteristics

ICU admissions encompass severe infections, acute respiratory failure, multiple organ dysfunction, major cardiovascular diseases, poisoning, drug overdose, and other serious life-threatening illnesses or trauma. These patients often present with complex conditions and may have underlying diseases such as diabetes, hypertension, chronic kidney disease, chronic liver disease, or abnormalities in the immune or respiratory systems. Clinicians managing these conditions may use sedative and analgesic medications like propofol, dexmedetomidine, benzodiazepines, and opioids, as well as steroid drugs for anti-inflammatory treatment (Stollings et al., 2022). Studies have found that patient-specific factors such as advanced age, frailty, diabetes, infection, a history of stroke, dementia or preoperative cognitive dysfunction, and medical factors such as general anesthesia and sedative use increase the risk of delirium in ICU patients (Chen et al., 2020). Additionally, fear and anxiety induced by the ICU environment and sleep disorders further elevate the incidence of delirium (Tronstad et al., 2021). Therefore, ICU delirium is closely related to the severity of the disease, specific medications, and the unique environment of the ICU. The use of environmental and physical constraints may act as triggers for psychological vulnerabilities (e.g., fear), but the impact of these triggers on groups with varying degrees of psychological vulnerability remains an important, unexplored topic.

## 3 Pathophysiological mechanism of ICU delirium

The precise mechanical trigger for ICU delirium remains unclear, and it is widely believed to be the result of a multifactorial interplay. The advancement of modern medicine has proposed several hypotheses to elucidate the etiology of this condition. We categorize and integrate six factors: neuroinflammation, neurotransmitter imbalance, brain function issues under pathological conditions, neuroendocrine disruption, oxidative stress, and gut microbiota imbalance. These factors interact with one another, particularly neuroinflammation, which influences the other factors and can contribute to delirium in the ICU, whether acting independently or in combination. Current clinical and pharmacological experimental studies have provided some support for the pathophysiological mechanisms of ICU delirium. This article hypothesizes potential reasons for the occurrence of delirium in critically ill patients, drawing from the phenomenology of delirium, and utilizes animal studies as well as cellular and molecular research to elucidate possible pathways. Finally, the article addresses some limitations and offers prospects for future research.

### 3.1 Neuroinflammatory pathways

**Figure 1:**
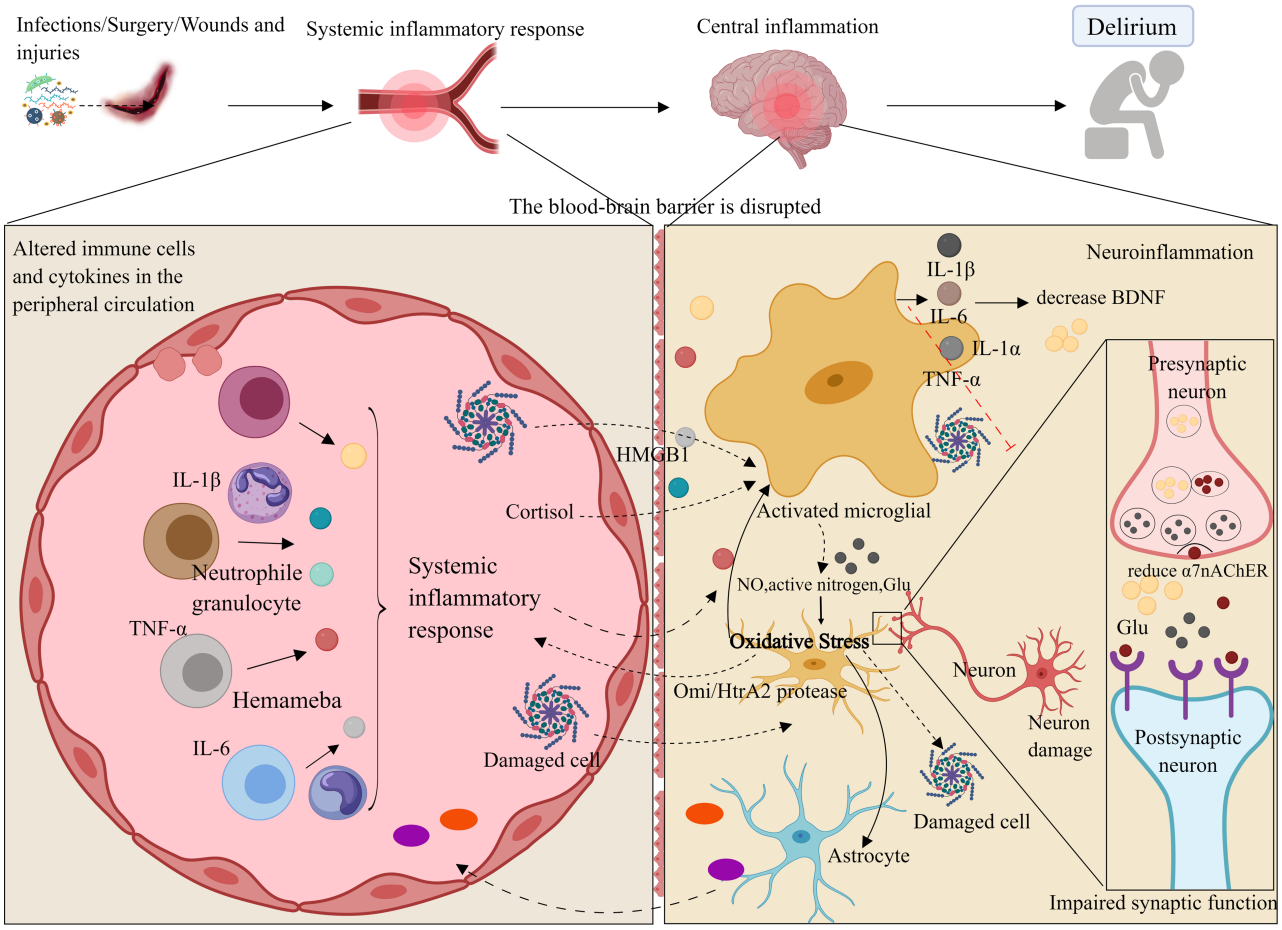
Neuroinflammation and oxidative stress. Neuroinflammatory pathways may compromise the blood–brain barrier through systemic inflammation, leading to the activation of brain parenchymal cells such as microglia and astrocytes by inflammatory factors (e.g., IL-1β, IL-6, TNF-*α*). Additionally, these pathways may disrupt neurotrophic factors via inflammatory responses, resulting in synaptic dysfunction or neuronal death, which can ultimately cause delirium and impair learning and cognitive functions. Oxidative stress may further contribute to delirium by decreasing antioxidant capacity and amplifying neuroinflammatory responses. ROS, Reactive oxygen species; NO, Nitric oxide; Omi/HtrA2, Serine protease. Images created using MedPeer (www.medpeer.cn).

Currently, numerous hypotheses propose that neuroinflammation contributes to the pathogenesis of delirium, with neuronal mitochondrial dysfunction, synaptic dysfunction, and neuronal death resulting from neuroinflammation being potential mechanisms underlying delirium (Kuperberg and Wadgaonkar, 2017; Xin et al., 2023). Previous studies have identified infection as a significant risk factor for ICU delirium, with systemic infections like sepsis potentially inducing brain dysfunction through inflammatory pathways (van den Boogaard et al., 2012). In major surgeries, such as cardiac surgery, ICU delirium has been linked to markers of inflammation present during and after the procedure (Su et al., 2023). A recent multicenter prospective observational study, after adjusting for potential confounders, found that higher levels of inflammatory markers (e.g., IL-6, IL-8, IL-10, TNF-*α*) are independently associated with delirium during critical ill (Brummel et al., 2024). Additionally, elevated levels of inflammatory markers and the glial activation-specific marker protein (S-100β) correlate with more prolonged and severe delirium in the ICU (Khan et al., 2020). These findings suggest that inflammatory factors may play a crucial role in the development of ICU delirium (see Figure 1).

When systemic inflammation occurs, inflammatory factors can promote inflammation of cerebral vascular endothelial cells via the bloodstream. This may damage the blood–brain barrier (BBB), allowing inflammatory factors to penetrate the BBB into the central nervous system, or disrupt brain function through stimulation of the vagus nerve efferent fibers and other trans-BBB mechanisms (Gonçalves and De Felice, 2021). This process can lead to the infiltration of peripheral inflammatory cells into the central nervous system. Direct observation of inflammation and specific pathways in the brains of delirium patients in the ICU is challenging. Animal studies have shown that, when mice are subjected to systemic inflammation and cognitive dysfunction, inflammatory factors in their hippocampus are significantly elevated (Wang H. C. et al., 2022). Another possible cause of delirium in the ICU is the activation of brain parenchymal cells (e.g., microglia and astrocytes) through various pathways, leading to impaired synaptic function of neurons. Increased expression of inflammatory mediators (e.g., TNF-*α*, IL-1β, IL-6, and HMGB1) may activate microglia and astrocytes, exacerbate neuroinflammation, enhance phagocytosis by brain parenchymal cells, impair excitatory synaptic function in the hippocampus, and reduce neuronal activity (Xin et al., 2023; Yin et al., 2023). This suggests a close relationship between neuroinflammation and neurotransmitter function. A reduction in α7 nicotinic acetylcholine receptor (α7nAChR) levels enhances microglial activity, which may promote the release of inflammatory factors, leading to neurotoxicity (van Gool et al., 2010; Xue et al., 2019). Furthermore, elevated levels of inflammatory factors can suppress brain-derived neurotrophic factor (BDNF) through the activation of the inflammatory signaling cascade, thereby compromising neuronal integrity (Felger and Lotrich, 2013; Dadkhah et al., 2024). In critically ill patients, vitamin B6, which has neurotrophic effects, is reduced during severe stress, inflammation, and disease exacerbation, large-dose vitamin B6 supplementation can enhance the immune response in these patients (Danielski et al., 2018; Cheng et al., 2006). Additionally, animal models indicate that interfering with the activation of the kynurenine pathway through vitamin B6 administration may reduce the incidence of delirium (Hou et al., 2012). This suggests that a deficiency in neurotrophic factors could also be the cause of delirium in critically ill patients.

Additionally, the systemic inflammatory response is associated with ICU-acquired weakness (ICU-AW), and studies have shown that inflammatory markers (e.g., IL-6, IL-8, IL-10) are elevated in patients with ICU-AW (Winkelman, 2010; Witteveen et al., 2017). Prolonged immobilization is a well-established risk factor for delirium. Inflammation can cause muscle fiber damage (Swash and de Carvalho, 2020) by releasing cytokines and chemokines that modulate the enzymatic response related to proteolysis. This results in loss of muscle strength, muscle atrophy, alterations in muscle function and structure, and a decrease in the patient’s mobility, thereby contributing to delirium (Marcantonio, 2017). Consequently, reducing inflammation and implementing early rehabilitation are crucial measures for preventing delirium in the ICU.

In conclusion, the neuroinflammatory pathway may compromise brain learning and cognitive function by disrupting the blood–brain barrier, stimulating brain parenchymal cells, and impairing neurotrophic factors, ultimately contributing to ICU delirium.

### 3.2 Neurotransmitter imbalance

**Figure 2:**
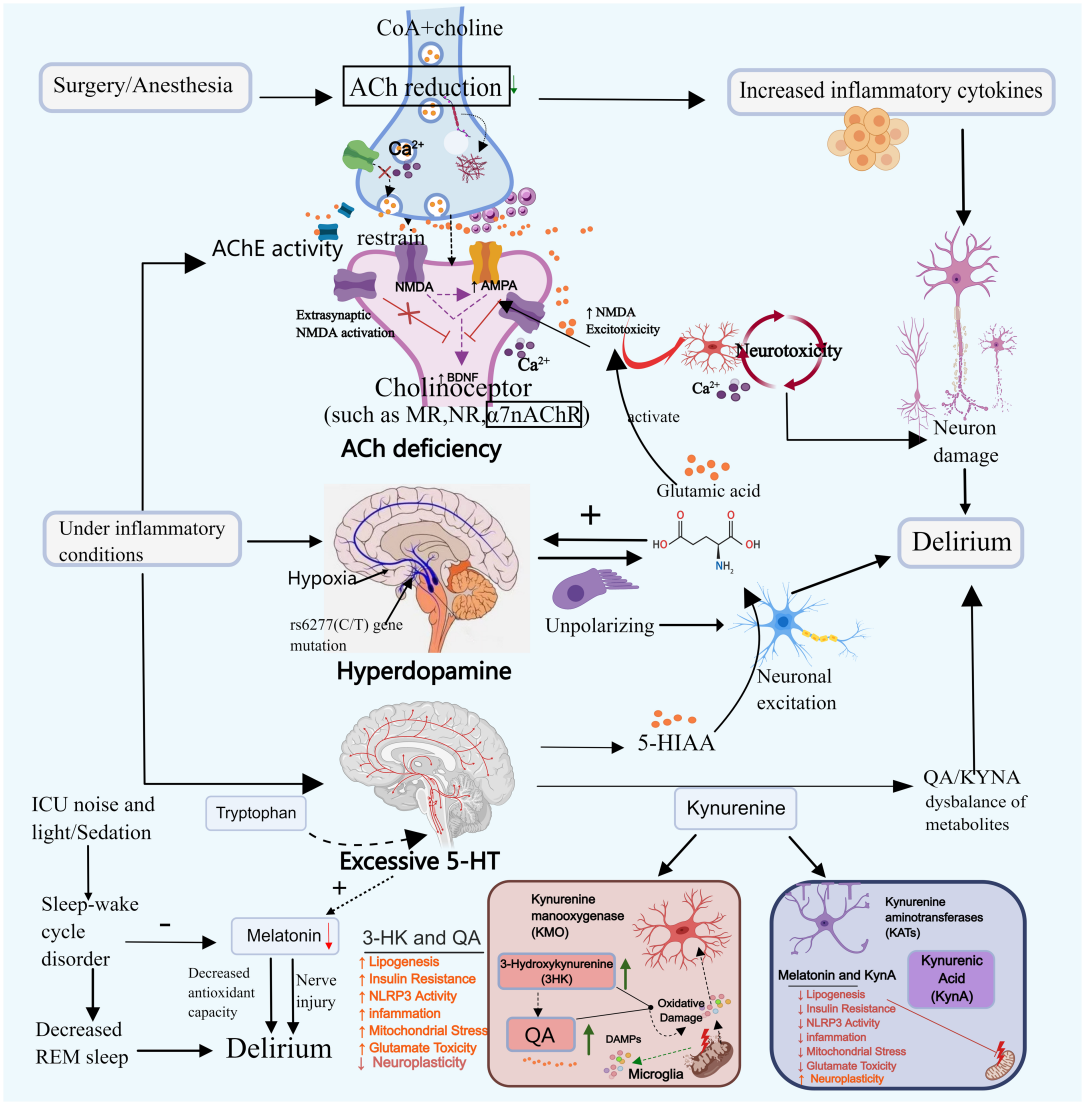
Neurotransmitter imbalance and sleep–wake cycle disturbance. Neurotransmitter imbalances may contribute to the onset of delirium. For instance, acetylcholine, dopamine, and serotonin can promote delirium through mechanisms such as reducing neural transmission, enhancing toxic responses, and decreasing melatonin secretion. Disruption of the sleep–wake cycle primarily triggers delirium by diminishing REM sleep and melatonin secretion. ACh, Acetylcholine; DA, Dopamine; 5-HT, Serotonin; CoA, Acetyl-coa; 5-HIAA, 5-hydroxyindoleacetic acid; TRP-KYN, Tryptophane-kynurenine; KP, Kynurenine pathway; QA, Quinolinic acid; KYNA, Kyinoquinolinic acid. Images created using MedPeer (www.medpeer.cn).

Neurotransmitter imbalances could represent another significant factor contributing to delirium, particularly concerning abnormal release, clearance, or altered receptor sensitivity of neurotransmitters. Neurotransmitters linked to delirium encompass acetylcholine (ACh), dopamine (DA), 5-hydroxytryptamine (5-HT), gamma-aminobutyric acid (GABA), norepinephrine, tryptophan, tyrosine, glutamate and so on. Inflammatory responses can impact neurotransmitter release. Pro-inflammatory cytokines such as TNF-*α*, IL-1β, and IL-6 modulate the neurotransmitter system by influencing the activity of enzymes implicated in neurotransmitter synthesis and modifying the expression of neurotransmitter transporters and receptors (Zahniser and Doolen, 2001). Among these, TNF-*α* has been demonstrated to stimulate a burst pattern of glial transmitter release that influences neuronal NMDA receptors (Lee and Haydon, 2011). Moreover, cytokines activate the kynurenine pathway, which not only depletes tryptophan, the precursor of serotonin, but also generates metabolites that can impact the modulation of dopamine and glutamate (Miller et al., 2013). This paper primarily discusses the causes of delirium related to cholinergic neurotransmitter deficiency and excess of monoaminergic neurotransmitters (see Figure 2).

#### 3.2.1 Deficiency of cholinergic neurotransmitter

Cholinergic neurons govern cognitive, learning, and memory-related cortical activity, and their systemic malfunction can induce delirium (Zheng et al., 2020). Animal experiments simulating delirium through scopolamine-induced cholinergic deficiency demonstrate that decreased ACh levels block the projection of cholinergic neurons to the hippocampus, leading to toxic reactions and resulting in cognitive and learning-memory impairments in mice (da Costa et al., 2022). Plasma acetylcholinesterase (AChE) and butyrylcholinesterase (BChE) activity can reflect cholinergic levels. Clinical studies have found that increased AChE activity is associated with delirium (Hughes et al., 2022). Cholinesterase inhibitors (AChEI) boost cholinergic activity and could potentially be utilized in the treatment of delirium. A retrospective cohort study revealed that the administration of Donepezil decreased the occurrence of delirium in patients with severe dementia (Lieberman et al., 2023b). However, since Donepezil is currently prescribed for mild to moderate Alzheimer’s dementia, direct evidence supporting its efficacy in treating delirium is lacking. Lieberman et al. (2023a) suggested the need for reevaluation of the role of AChEI in the prevention and treatment of delirium during critical illness. In addition to AChE and BChE, the study found that serum anticholinergic activity (SAA) levels were significantly elevated in patients with critical infections. This increase was closely associated with the occurrence of delirium, providing an alternative reflection of cholinergic levels and further confirming the correlation between decreased cholinergic levels and ICU delirium (Zhao et al., 2020; Liu et al., 2017).

ACh is synthesized through the collaborative action of coenzyme A (CoA) and choline, and any disruption in this pathway can lead to a decrease in ACh levels. Primarily derived from the breakdown of glucose, fats, and amino acids, CoA may contribute to reduced ACh levels in cases of severe malnutrition or hypoglycemia. In early severe infection, AChE translocates from plasma to the tissue space, leading to low serum cholinesterase levels in infected patients, this is associated with its decreased expression at the neuromuscular junction and oxidative stress. Subsequently, the body expends a significant amount of ACh to activate the cholinergic anti-inflammatory pathway and alleviate the inflammatory response (Liu et al., 2017; Xia et al., 2022).

Impaired synaptic function of ACh may serve as a significant contributing factor to the development of delirium. The ACh binds to the muscarinic receptor (M receptor) to exert its effects. Research indicates that surgical procedures or anesthesia can harm the M receptor in hippocampal astrocytes, leading to synaptic dysfunction and subsequent cognitive decline. Nevertheless, following the administration of galantamine to enhance ACh release, cells can regain normal signal transduction, fostering cognitive enhancement (Wang T. et al., 2022). Initial studies have demonstrated that certain anesthetics (such as isoflurane, pentobarbital, and chloral hydrate) can diminish ACh levels and impede postsynaptic nicotinic receptors. Conversely, dexmedetomidine may mitigate delirium by elevating ACh levels in the hippocampus (Damsma and Fibiger, 1991; Zhang et al., 2016). Clinical trial findings have additionally demonstrated that prophylactic administration of low-dose dexmedetomidine can significantly decrease the incidence of postoperative delirium within 7 days among patients admitted to the ICU following non-cardiac surgery (Su et al., 2016). These results require further investigation. Nevertheless, there have also been trials yielding negative results. Furthermore, cholinergic deficiency exhibits a synergistic effect with neuroinflammation. Reduced ACh levels enhance the secretion of IL-1β, IL-6, TNF-αand other inflammatory markers (Saeed et al., 2005), thereby exacerbating the inflammatory response. Within the ACh system, α7nAChR serves a neuroprotective and cognitive function. It hinders the production of pro-inflammatory cytokines by inhibiting nuclear factor-kappa B (NF-κB) signaling and orchestrates immune responses by directing macrophages towards inflammatory sites (Keever et al., 2024). Nevertheless, α7nAChR levels were observed to be lower in the delirium group compared to the control group, suggesting that ACh deficiency may induce delirium by suppressing α7nAChR expression. This inhibition can lead to heightened release of inflammatory factors, activation of the NF-κB, Phosphatidylinositol 3 kinase/protein kinase B(PI3K/AKT), and Glycogen synthase kinase 3β (GSK-3β) pathways, and exacerbation of neuronal and cellular apoptosis (Xue et al., 2019; Wang et al., 2018). Inflammation can further diminish ACh release, AChE activity and expression can be regulated in a cell-specific manner through the binding of DNA binding sites with NF-κB and other transcription factors involved in the inflammatory response (de Oliveira et al., 2012). In the presence of inflammation, AChE activity may increase, subsequently leading to a reduction in ACh levels, thereby establishing a detrimental cycle between the two.

Most studies on ACh in the hippocampus are based on animal experiments, and the mechanisms may differ in humans. Clinical trials involving AChEI have shown diverse outcomes, with one study revealing higher delirium duration, severity, and mortality in the carbamate group compared to the placebo group (van Eijk et al., 2010), potentially attributed to significant differences in baseline characteristics between the two groups. Further investigation into the role of AChEI in delirium is warranted.

#### 3.2.2 Excessive monoaminergic neurotransmitters

##### 3.2.2.1 Excessive dopamine (DA)

DA stands as the predominant catecholamine neurotransmitter in the brain, governing hippocampal synaptic plasticity and bearing significance in cognitive functions, memory, and more. The concentration of its metabolite, homovanilic acid, is associated with delirium in patients exhibiting psychotic symptoms, such as hallucinations and delusions (Tsetsenis et al., 2022; Ramirez-Bermudez et al., 2008). Moya et al. (2023) discovered that an overabundance of dopamine release in the dorsal striatum could result in cognitive-related behavioral impairments in mice. Drugs such as benzylpiperazine and amphetamine enhance dopaminergic neurotransmission and can induce clinical manifestations of delirium, including agitation, confusion, and hallucinations (Schep et al., 2011; Chien-Po and An-Yi, 2022). This suggests that excessive DA may also contribute to the development of delirium.

Tetrahydrobiopterin (BH4), a crucial enzyme cofactor in dopamine synthesis, is under the influence of inflammatory mediators. Factors such as IL-1β, interferon gamma (IFN-*γ*), and TNF-*α* may elevate BH4 levels, subsequently enhancing dopamine production (de Bartolomeis et al., 2022). In instances of acute hypoxia in patients, dopamine levels may surge in response to acute stress. Furthermore, as cerebral ischemia and hypoxia worsen, DA release is likely to escalate (Dong et al., 2024; Oliva et al., 2013). DA exhibits a specific correlation with ACh. Dopamine receptors can be categorized into D1-like receptors (comprising D1 and D5) and D2-like receptors (comprising D2, D3, and D4). Activation of D1-like receptors facilitates the breakdown and conversion of ACh, whereas activation of D2-like receptors can enhance ACh synthesis (Guidolin et al., 2023). D1-like receptors heighten their activity in reaction to heightened intracellular cyclic adenosine monophosphate (cAMP) levels, depolarized membrane potential, and increased dopamine availability. This elevation leads neurons to an excited state, contributing to the manifestation of delirium symptoms. DA and glutamate engage in mutual interactions. Past research indicates that elevated levels of DA can induce nerve cell membrane depolarization, triggering substantial glutamate production. This excess glutamate can subsequently modulate dopaminergic transmission in the striatum, thereby promoting a selective enhancement of DA release (Bleck, 2018; Rudolph et al., 1983). Disrupted glutaminergic signaling, facilitated by sufficient depolarization of the postsynaptic membrane and inhibition by Mg2+-removing factors, can mildly and persistently stimulate the N-methyl-D-aspartate glutamate receptor (NMDAR). This stimulation leads to prolonged influx of Ca2+ into postsynaptic neurons. Excessive Ca2+ influx may culminate in the gradual impairment of dopaminergic mesencephalic and cortical synaptic function, ultimately resulting in neuronal demise (Chang et al., 2020; Antonelli et al., 2007). Additional studies have proposed a connection between certain genes in the dopaminergic system and the onset of delirium. Specifically, the rs6277 CC genotype has been linked to delirium occurrence in ICU patients. Cell studies suggest that the rs6277 (C/T) gene mutation disrupts the balance between DA and ACh by heightening dopaminergic neural activity, potentially elevating the risk of delirium (Li, 2023).

Nonetheless, there are divergent views on this hypothesis. Henjum et al. (2021) discovered that the cerebrospinal fluid dopamine levels of delirious hip fracture patients were lower than those of non-delirious patients. Additionally, there was no significant difference between the effectiveness of dopamine antagonist (haloperidol) and placebo in treating ICU delirium (Andersen-Ranberg et al., 2022). Therefore, the notion that excessive DA causes delirium remains a topic open to debate.

##### 3.2.2.2 Excessive 5-HT

Research has demonstrated elevated serotonin levels in patients experiencing delirium (Egberts et al., 2015). An observational prospective cohort study revealed that frequent and prolonged administration of drugs with serotonergic properties in ICU patients led to central serotonin overdoses, which in turn resulted in delirium (van Ewijk et al., 2016). Conversely, ICU patients who were treated with serotonin reuptake inhibitors had a lower risk of delirium within 24 h of medication compared to those in the control group (Austin et al., 2022).

5-HT may influence the development of delirium primarily through its metabolites. Studies have shown that the level of 5-hydroxyindoleacetic acid (5-HIAA), the main metabolite of 5-HT, is higher in delirium patients compared to non-delirium patients (Heylen et al., 2022). This increase in 5-HIAA may potentially exacerbate delirium by enhancing excitatory neurotransmission. In animal experiments, the local administration of 5-hydroxyindole significantly increased glutamate concentration in the hippocampus, resulting in neurotoxicity that affects brain memory and cognitive function (Mannaioni et al., 2003). Another metabolite, kynurenine, is also linked to delirium. Elevated levels of 5-HT increase the concentration of kynurenine, consequently increasing the incidence of delirium significantly (Adams Wilson et al., 2012). Studies indicate that the buildup of neurotoxic metabolites in the tryptophan-kynurenine (TRP-KYN) pathway plays a role in the onset of delirium (Voils et al., 2020). This pathway is associated with inflammation, and when pro-inflammatory cytokine levels are elevated in critically ill patients, the kynurenine pathway (KP) generates neuroactive metabolites: Quinolinic acid (QA) and Kynurenic acid (KYNA). QA is neurotoxic, while KYNA has a neuroprotective effect. In the case of acute inflammation (such as trauma, surgery, and sepsis), the imbalance between neuroprotective and neurotoxic metabolites in the kynurenine pathway may result in neuronal damage and delirium (Phing et al., 2023).

### 3.3 Abnormal brain function under neuropathological conditions

#### 3.3.1 Abnormal functional connectivity of the brain

Brain functional connectivity, which establishes a functional network that controls the body, transmits information between regions of the brain and plays a significant role in the onset of delirium. Functional connectivity refers to statistical correlations between neurophysiological data recorded in various brain regions. It reflects the capacity of brain to synchronize activity across spatially distributed regions, which is essential for coordinated information processing to support cognitive function. Functional connectivity is commonly assessed using neuroimaging techniques such as functional magnetic resonance imaging (fMRI) and electroencephalography (EEG) (Litwińczuk et al., 2022; Voigt et al., 2023). Analysis indicates that delirium consistently correlates with slower EEG patterns and reduced functional connectivity. These findings are characterized by decreased alpha-band EEG connectivity and diminished integration within fMRI networks (Boord et al., 2021; van Montfort et al., 2019). Factors predisposing to delirium, such as aging, were linked to reduced fMRI connection strength. Triggering factors, such as the use of sedatives, were associated with decreased overall functional connectivity strength, alpha-band network efficiency, and regional functional connectivity between the posterior cingulate gyrus and dorsolateral prefrontal cortex (van Montfort et al., 2019; van Montfort et al., 2020). A modeling study (Ponten et al., 2013) proposed that an imbalance in the activity of excitatory and inhibitory neurons, along with heightened subcortical information fluctuations, could result in functional brain connectivity dysfunction, ultimately contributing to delirium.

Neurons at various levels form interconnected networks and interact with one another. In cases of reduced cerebral blood perfusion, cerebral hypoxia, or cerebral microembolism in patients, disruptions in brain metabolism, hemodynamics, and network function may arise, leading to functional neuroimaging alterations. These changes may manifest as decreased blood flow ratios in the left inferior frontal lobe, right temporal lobe, and the right brain region. The default mode network (DMN) is a system that links various brain regions. In states of delirium, the direction of functional connections between the posterior cingulate cortex and the dorsolateral prefrontal cortex is reversed (Haggstrom et al., 2017). Apart from the default mode network (DMN), delirium may lead to varying degrees of dysfunction in resting neural networks. Moreover, individual variability in brain structures, such as chronic cerebrovascular changes in the elderly, can also influence the directionality of functional connections (Hanna et al., 2023). The Ascending Reticular Activation System (ARAS) is a network of neurons located in the brainstem regulating wakefulness, sleep, and dreaming states via connections to the suprachiasmatic nucleus (SCN) and the release of acetylcholine during wakefulness. Nevertheless, in cases of delirium, ARAS activity diminishes, leading to neurotransmitter imbalances and sleep–wake disturbances that contribute to the onset of delirium (Hut and Van der Zee, 2011).

#### 3.3.2 Disturbed sleep–wake cycle

Research indicates that patients in the ICU experiencing delirium exhibit a lower frequency of Rapid Eye Movement (REM) periods compared to those without delirium (Sun et al., 2021). REM sleep is characterized by rapid eye movements, brain activity resembling a waking state, and is typically accompanied by vivid dreams. It is considered crucial for memory consolidation and emotional regulation. Moreover, individuals with delirium commonly display disrupted sleep–wake cycles. Exposure of subjects to simulated ICU noise and light environments often leads to sleep deprivation and fragmentation; however, when noise and light levels are reduced, their sleep patterns tend to normalize (Hu et al., 2010). In the ICU, the administration of sedatives, particularly benzodiazepines, can disrupt the regular sleep–wake cycle by promoting lighter sleep and reducing slow-wave and REM sleep stages (Devlin et al., 2018). Sedation is a recognized confounder in delirium screening, where a patient with an RASS (a scale used to assess sedation and agitation in ICU patients) is less than −2, the level of arousal may influence the assessment of delirium. Within this score range, patients exhibit very limited responses to stimuli, which may result in delirium symptoms being masked or inaccurately evaluated. Distinguishing between sedation and delirium is crucial as they can lead to a positive screening and a similar “delirium diagnosis.” Hence, it is essential to not only evaluate the sedative type but also consider the frequency of sedative administration in critically ill patients to minimize confounding variables in delirium assessments. Research has indicated that the metabolism of sedatives is influenced by inflammation, where inflammatory mediators (such as IL-6 and TNF-*α*) are associated with the suppression of cytochrome P450 enzyme (CYP) expression and activity. This leads to elevated blood drug concentrations and subsequent alterations in drug efficacy (Abdallah et al., 2023). Furthermore, the disruption of the blood–brain barrier (BBB) by inflammatory mediators can potentially enhance the toxicity of sedatives.

The Society for Critical Care Medicine (SCCM) acknowledges the correlation between sleep and the development of delirium in critically ill adults; however, a causal relationship has not yet been definitively established (Devlin et al., 2018). Disruptions in the sleep–wake cycle can potentially precipitate delirium by causing imbalances in neurotransmitters and disrupting the rhythmic secretion of endogenous melatonin. These disturbances may impact the synthesis, release, and inactivation of crucial neurotransmitters like ACh and DA, ultimately playing a role in the development of delirium (Figueroa-Ramos et al., 2009). Research indicates a significant correlation between melatonin levels and delirium in critically ill patients. Melatonin has the capacity to modulate circadian rhythms and act as an antioxidant, thereby positively impacting the body. However, in patients with delirium, the peak secretion of melatonin occurs during the day, disrupting the typical diurnal fluctuation of lower levels during the day and higher levels at night (Yoshitaka et al., 2013; Sun et al., 2014). One significant factor contributing to altered melatonin levels in critically ill patients is encephalopathy or brain dysfunction, which can hinder and prolong the amplitude and peak timing of melatonin secretion. Moreover, atypical light exposure, irregular nutrient intake, and heightened arousal in the ICU environment often disrupt the circadian rhythm, subsequently impacting melatonin secretion. Furthermore, the development of severe sepsis in patients can further impair the circadian rhythm of melatonin secretion (Maas et al., 2020; Mundigler et al., 2002). A meta-analysis revealed a 34% reduction in the incidence of delirium among critically ill patients following the administration of exogenous melatonin, demonstrating a significant preventive effect on delirium (Khaing and Nair, 2021; Zhang Q. et al., 2019). Melatonin influences the sleep–wake cycle by activating two high-affinity G-protein-coupled receptors, MT1 and MT2 (Liu et al., 2016). Through its actions, melatonin can suppress the activity of hypocretin neurons via MTR1, dampen the excitability of suprachiasmatic nucleus (SCN) neurons and glutamatergic neurons in the paraventricular nucleus of the thalamus, and stimulate gamma-aminobutyric acid (GABA) ergic neurons in the reticular nucleus of the thalamus, facilitating the transition from wakefulness to sleep (Sharma et al., 2018; Yue, 2019). Disrupted secretion patterns of melatonin can alter the sleep–wake cycle, influencing the transition between sleep and wakefulness or the equilibrium between the two states.

Divergent opinions persist regarding the secretion and clinical impacts of melatonin. Wibrow et al. (2022) conducted a randomized controlled trial that revealed no reduction in delirium prevalence in patients initiating melatonin within 48 h of ICU admission. In a separate study, Simons et al. (2016) employed bright light therapy during daytime to modulate circadian rhythms but observed no significant variations in melatonin levels, cumulative delirium incidence, or duration in ICU-acquired delirium between the treatment and control groups. Consequently, further extensive, multicenter investigations are warranted to elucidate whether disruptions in the sleep–wake cycle and melatonin levels indeed influence delirium onset.

#### 3.3.3 The disease directly causes brain function impairment

Direct brain damage encompasses both systemic and localized energy disorders (e.g., hypoglycemia, stroke), metabolic abnormalities (e.g., hyponatremia, hypercalcemia), and drug-induced effects (Maclullich et al., 2008). Glucose serves as the primary energy source for the brain to sustain the body’s normal functioning. In cases of hypoglycemia, where patients experience low blood glucose levels, the brain is deprived of adequate glucose, leading to neurological symptoms of hypoglycemia, such as cognitive dysfunction and behavioral alterations (Blonde et al., 2022). While the impairment of brain function in ischemic stroke (IS) is typically permanent rather than a fluctuating cognitive deficit, it remains crucial not to overlook this condition. Research indicates that the incidence of delirium in the acute phase of IS is 14.8%. MRI findings suggest that IS patients with infarctions in the left cortical region are more susceptible to delirium, possibly due to the role of the dominant hemisphere, particularly the limbic system and the prefrontal cortex, in regulating emotions, memory, and cognitive functions. Infarctions in this area directly contribute to brain function impairment. Moreover, distinct features of IS can result in various subtypes of delirium (Qu et al., 2018). The pathophysiology of IS involves a series of energy pump failures and intricate signaling cascades that culminate in neuronal cell death. Dysfunction of the brain’s energy pump results in impaired brain oxidative metabolism. The ischemic cascade entails heightened glutamatergic transmission, leading to excessive glutamate release, augmented intracellular sodium and calcium influx, free radical generation, and the release of inflammatory cytokines. These processes ultimately result in cell edema, cell death, and subsequently impact the signal propagation in the cerebral cortex (Xu et al., 2020; Gulyaeva et al., 2021). In the event of IS, the body swiftly generates hypoxia-inducible factor-1α (HIF-1α), which exerts a dual impact on neurons and microglia. While HIF-1α offers protective effects on nerve function, it can exacerbate blood–brain barrier (BBB) disruption and other stroke symptoms by elevating vascular endothelial growth factor (VEGF) within 1 h post-cerebral ischemia. The Mixed Lineage Kinase Domain-Like Protein (MLKL)/Receptor Interacting Protein 3 (RIP3) complex and the Notch pathway enhance apoptosis, worsening neuronal injury by activating microglia to heighten neuroinflammation and upregulate toll-like receptor 4 (TLR4) expression (Vatte and Ugale, 2023). IS may also play a role in post-stroke cognitive and emotional impairment by increasing cortisol levels, potentially resulting in hippocampal damage (Gulyaeva et al., 2021). Hyponatremia can induce delirium through direct neurotoxic effects and indirect alterations in brain volume and neuronal function. It can elevate extracellular glutamate levels in the hippocampus, leading to neurotoxicity. Furthermore, hyponatremia can trigger cerebral edema and heightened intracranial pressure, presenting as changes in neurological status and delirium (Fujisawa et al., 2016; Evanson and Espiridion, 2023). On the other hand, hypercalcemia may disrupt neurotransmitter balance by stimulating glutaminergic, dopaminergic, and serotonergic pathways, consequently causing delirium (Nagy et al., 2020).

### 3.4 Neuroendocrine disorders

**Figure 3:**
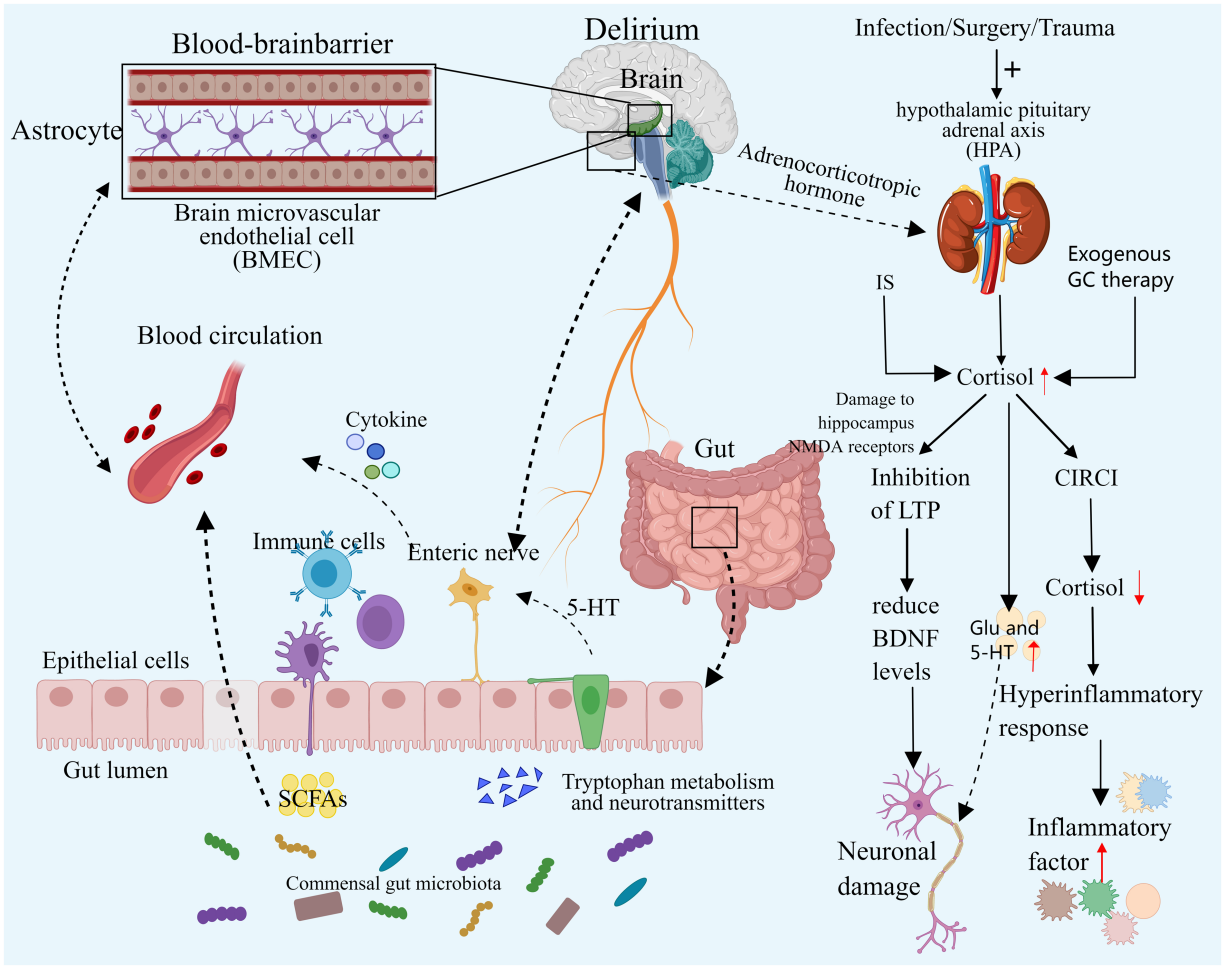
Neuroendocrine disorders and intestinal flora disorders. Both elevated and decreased cortisol levels can contribute to the development of delirium. Excessive cortisol may exacerbate neuroinflammation, resulting in neuronal damage, and it may also impair synaptic function and disrupt neurotransmitter balance, ultimately leading to delirium. In cases of severe infection, secondary CIRCI can result in decreased cortisol levels that fail to adequately control the inflammatory response, thereby inducing delirium. Additionally, an imbalance in the gut microbiome may contribute to delirium by altering microbial diversity and composition, directly affecting specific gut bacteria, and exacerbating neuroinflammation and neurotransmitter imbalances. IS, Ischemic stroke; GC, Glucocorticoid; HPA, Hypothalamic–pituitary–adrenal cortex; CIRCI, Critical illness associated corticosteroid insufficiency; DSV, Desulfovibrio Bacteria; SCFA, Short-chain fatty acids, EC, Enterochromaffin cells. Images created using MedPeer (www.medpeer.cn).

Studies have revealed that abnormal hormonal fluctuations during stress, particularly disrupted cortisol levels, are associated with the onset of delirium. However, the precise mechanisms underlying this link and how these hormonal abnormalities trigger delirium remain incompletely understood. The variations in glucocorticoids (GC), melatonin, and catecholamines in the serum or cerebrospinal fluid of ICU patients are intricately linked to delirium onset. Certain hormones function as neurotransmitters, exerting targeted actions on specific cells and eliciting diverse physiological effects (Cao et al., 2023). This section specifically delves into the impact of glucocorticoids on delirium (see Figure 3).

Prior studies have indicated elevated levels of cortisol in the CSF and plasma in delirious patients, with increased cortisol levels serving as a predictor for delirium occurrence in specific subgroups of critically ill patients (Pearson et al., 2010; Bisschop et al., 2011; Michels et al., 2019).

During intense stress, the hypothalamic–pituitary–adrenal cortex (HPA) axis undergoes temporary activation, leading to increased synthesis and secretion of adrenal cortisol through stress-induced enhancement mechanisms. Notably, this process involves the inhibition of peripheral cortisol metabolism, resulting in reduced plasma binding of cortisol and suppression of metabolic enzyme expression and activity in the liver and kidney. Consequently, cortisol breakdown is diminished, leading to elevated blood cortisol levels. Subsequently, the elevated plasma cortisol concentration triggers a negative feedback loop, reducing plasma adrenocorticotropic hormone (ACTH) levels. This cascade can result in critical illness-associated corticosteroid insufficiency (CIRCI), characterized by normal or even subnormal cortisol levels (Martinez et al., 2024; Langouche et al., 2023; Boonen et al., 2013; Boonen and Van den Berghe, 2016). Simultaneously, critically ill patients may receive exogenous glucocorticoid therapy, known for its anti-inflammatory and immunomodulatory effects. Current research advocates for the judicious use of hydrocortisone in critically ill patients. Nevertheless, prolonged or excessive glucocorticoid administration can induce delirium symptoms such as apathy, restlessness, and disorientation (Langouche et al., 2023; Yang et al., 2021). A substantial retrospective cohort study indicated a potential exacerbation of delirium with dexamethasone use in critically ill patients, with a dose-dependent increase in delirium risk (Wu et al., 2021).

Previous studies have demonstrated a significant correlation between cortisol levels and delirium in critically ill patients with infections and sepsis (van den Boogaard et al., 2011; Pfister et al., 2008). Stress triggers activation of the HPA axis, leading to heightened cortisol synthesis and delirium induction. While cortisol can exert an anti-inflammatory effect by inhibiting pathways such as NF-κB and Janus kinase signal transduction and transcriptional activator pathway (JAK–STAT) (Sarapultsev et al., 2023), it may, under specific circumstances, enhance the expression of inflammatory mediators or boost inflammatory cell activity, exacerbating the inflammatory response, especially during stress responses. In severe infections, inadequate cortisol levels can result in an exaggerated inflammatory response, heightening neuroinflammation and elevating the risk of delirium onset. Human experiments have shown that transient elevation of cortisol levels to stress levels correlates with increased systemic inflammatory responses (Yeager et al., 2009). In animal studies, exposing mouse microglia to varying cortisol (CORT) concentrations revealed that excessive CORT, particularly under low temperatures, activated glia, exacerbating neuroinflammation in the Dentate Gyrus (a key component of the hippocampal structure, is situated in the central region of the hippocampus) through HMGB1 acetylation and enhanced NF-κB signaling (Xu et al., 2019). In a simulated neuroinflammation delirium model, reducing cortisol levels with medication can reverse attention and learning deficits (Carr et al., 2019), further demonstrating the harmful role of excessive cortisol in neuroinflammation-induced delirium. Therefore, both excessively low and high cortisol levels may precipitate delirium. Elevated levels of glucocorticoids may also contribute to delirium by influencing synaptic function in neurons. Animal studies have revealed a common mechanism of action in the hippocampus, where acute high concentrations of corticosterone and chronic stress can impair the hippocampal NMDA receptor by activating the glucocorticoid receptor, suppressing the occurrence of long-term potentiation (LTP), and ultimately diminishing levels of brain-derived neurotrophic factor (BDNF) in the hippocampus, a crucial component for neuronal function in the cortex and striatum. Consequently, high levels of glucocorticoids could affect the limbic system, potentially leading to delirium (Park et al., 2015; Jin et al., 2019). Additional studies have demonstrated that GC activates GSK-3 through Akt1 regulation, impeding synaptic signaling, inhibiting LTP, and inducing tau protein phosphorylation, ultimately impairing neuronal synaptic function (Yi et al., 2017). Elevated cortisol levels may also promote delirium by disrupting neurotransmitter balance. Stress-induced elevated GC may lead to neurotoxicity by boosting intracellular Ca2+ and glutamate synaptic transmission. Furthermore, excessive GC can amplify 5-HT activity, which also contributes to delirium (Prager and Johnson, 2009; Joëls and Van Riel, 2004).

### 3.5 Oxidative stress pathway

Prior studies have indicated that patients experiencing oxidative stress may develop delirium (Seaman et al., 2006). Under normal circumstances, cells rely on diverse defense mechanisms to shield themselves against reactive oxygen species (ROS). However, during periods of stress or inflammation, an imbalance arises between ROS production and the body’s antioxidant defense mechanisms. Excessive ROS then contribute to the oxidation of biological macromolecules, leading to cellular lipid peroxidation and mitochondrial dysfunction, culminating in oxidative stress (Pang et al., 2022). In critically ill patients, conditions like tissue ischemia–reperfusion injury and systemic inflammatory responses, as seen in sepsis and acute respiratory distress syndrome, can worsen the disparity between oxidation and antioxidation. This can lead to heightened production of reactive oxygen species (ROS) and other free radicals, or an elevation in lipid peroxidation product levels, ultimately resulting in a reduction in overall antioxidant capacity (Crimi et al., 2006; Bar-Or et al., 2015; Alonso de Vega et al., 2002).

Oxidative stress pathways can potentially trigger delirium by diminishing the endogenous antioxidant capacity. Randomized controlled trials have demonstrated that melatonin can enhance the overall antioxidant capacity in critically ill patients. Melatonin is capable of directly scavenging hydroxyl, peroxygen, and other free radicals. Moreover, it may act as an antioxidant by forming complexes with metal ions, repairing oxidative molecular damage, and stimulating antioxidant enzymes (Mistraletti et al., 2017; Purushothaman et al., 2020; Sieminski et al., 2023). Nevertheless, mechanical ventilation or the administration of commonly used ICU medications like benzodiazepines, steroids, anti-infectives, anticonvulsants, etc., can suppress melatonin secretion (Bourne and Mills, 2004), consequently diminishing its antioxidant capacity. Furthermore, oxidative stress exhibits a synergistic relationship with inflammation and neuroinflammation. Markers of oxidative dysfunction, such as sepsis and pneumonia, occur more frequently in patients diagnosed with delirium (Seaman et al., 2006). Animal studies have shown that the severity of sepsis correlates with persistent oxidative stress in the brain (de Souza et al., 2020). Neuroinflammation can increase mitochondrial oxidative stress and lead to a loss of mitochondrial membrane potential through the pathological activation of dynamin-related protein 1, ultimately resulting in mitochondrial damage and neuronal death (Haileselassie et al., 2020). Following the activation of neuroinflammatory microglia, the release of nitric oxide (NO), reactive nitrogen species, and glutamate can further cause structural damage to cell membranes and elicit an inflammatory response (Yan et al., 2022). Clinical observational studies have revealed a significant reduction in the oxidative stress index catalase (CAT), reflecting antioxidant capacity, in patients with postoperative delirium (Karlidag et al., 2006). Animal studies indicate a decrease in postoperative brain-derived neurotrophic factor (BDNF) expression, disrupting mitochondrial fission/fusion dynamics in the mouse brain and impairing mitochondrial function, thus contributing to delirium (Lu et al., 2020). The serine protease Omi/HtrA2, known for its proapoptotic properties within mitochondria, may facilitate inflammation-induced apoptosis and BBB disruption via caspase-dependent apoptotic pathways, ultimately leading to cognitive decline (Hu et al., 2019). Moreover, micronutrient deficiencies exacerbate inflammatory oxidative stress, correlating with a higher prevalence of delirium (Ceolin et al., 2023). In essence, oxidative stress likely plays a role in delirium development by diminishing antioxidant capacity and amplifying neuroinflammatory responses.

### 3.6 Imbalance of intestinal flora

Research has indicated that the incidence of ICU delirium is linked to gastrointestinal dysfunction stemming from an imbalance in intestinal flora. Patients with ICU delirium exhibit reduced diversity in intestinal flora and heightened intestinal barrier damage (Jiang, 2022; Gao, 2021).

Patients admitted to the ICU may experience ischemic events such as hemorrhage and hypotension, leading to ischemia redistribution and intestinal mucosal ischemia, which significantly contribute to intestinal flora imbalance. Moreover, ICU patients commonly face intestinal nutritional deficiencies and various invasive procedures that compromise the integrity of the intestinal epithelial barrier. The critical condition itself can induce increased intestinal barrier permeability and alterations in epithelial cells, leading to bacterial translocation. Furthermore, the administration of antibiotics, proton pump inhibitors, and opioids can directly impact the gut microbiota, with inappropriate antibiotic use being linked to the emergence of multidrug-resistant bacteria (Moron et al., 2019; Zhang J. et al., 2019).

The intestinal microbiota influences the host’s behavior by modulating flora and generating metabolites along the bidirectional microbial-gut-brain axis, impacting the nervous system and modulating brain signals (Shen and Sun, 2021; Rutsch et al., 2020). Imbalance in the gut microbiota can disrupt the diversity and composition of gut bacteria, with specific strains linked to delirium development. Research indicates that greater *α* diversity (higher microbial abundance and diversity) correlates with a reduced delirium risk, while patients with delirium exhibit a higher presence of Enterobacteriaceae genera linked to pro-inflammatory pathways (Garcez et al., 2023). A recent analysis of 211 gut microbiota using Mendelian randomization revealed that the presence of Desulfovibrio bacteria (DSV) and the species Candidatus Soleaferrea increases the risk of delirium. Conversely, the genera Oxalobacteriaceae, Holdemania, *Ruminococcus gnavus*, and Eggerthella reduce the risk of delirium (Yu et al., 2023). Desulfovibrio bacteria produce excessive hydrogen sulfide, which can breach the intestinal mucosa. At lower concentrations, this can impact neuronal signal transmission, while at higher concentrations, it can lead to toxic reactions in mitochondria, subsequently affecting the brain (Earley et al., 2015; Haouzi et al., 2020). An imbalance in intestinal flora can exacerbate the inflammatory response and intensify neuroinflammation. Dysregulation of the intestinal flora can result in elevated lipopolysaccharide (LPS) levels and activation of Toll-like receptor 4 (TLR4), triggering inflammatory signaling pathways associated with cognitive impairment (Narabayashi et al., 2022; Li et al., 2023). Animal studies have demonstrated that probiotics can regulate dysregulated gut flora and the microbial-brain-gut axis, thereby mitigating blood–brain barrier (BBB) permeability, reducing oxidative DNA damage, and inhibiting the NF-κB signaling pathway mediated by TLR4 and retinoid-inducing gene protein I (RIG-I) (Yang et al., 2020). Inflammation can disrupt the gut microbiota. Research analysis has revealed a correlation between the severity of sepsis in patients and the extent of intestinal flora imbalance (Liang et al., 2023). Short-chain fatty acids (SCFAs), produced by intestinal microorganisms, play a crucial role in regulating intestinal inflammation and reshaping intestinal ecology (McHardy et al., 2013). Animal models have shown that sepsis reduces SCFA levels, impacting brain function via the gut-microbiome-brain axis and leading to cognitive impairment (Zhang et al., 2023; Giridharan et al., 2022). The gut microbiota can influence the development of delirium through neurotransmitter regulation. Delirium patients exhibit higher levels of Serratia, Bacteroides, and Paracoides, with Serratia linked to DA and the latter two linked to GABA (Garcez et al., 2023). Additionally, research indicates that the intestinal microbiota can communicate directly with intestinal chromaffin cells (EC) via soluble local spore-forming bacteria, stimulating endogenous and local 5-HT biosynthesis, thereby affecting the nervous system (Yano et al., 2015).

In essence, gut flora dysregulation may contribute to delirium by altering flora diversity and composition, interacting with specific gut bacteria, intensifying neuroinflammation, and disrupting neurotransmitter balance.

## 4 Prospect

Future research should further explore the specific pathways and interconnections of the aforementioned mechanisms. More high-quality studies, such as randomized controlled trials and large-scale cohort studies, are needed to validate these mechanisms and their specific roles in the development of delirium, translate basic research findings into clinical applications, and develop prevention and treatment strategies for delirium based on these mechanisms. Exploring novel pathophysiological mechanisms like genetic factors, mitochondrial dysfunction, and endoplasmic reticulum stress can provide a comprehensive understanding of delirium mechanisms. Furthermore, identifying specific biomarkers for predicting ICU delirium stands as a crucial area of ongoing research.

## 5 Conclusion

Delirium is a common complication in critically ill patients, and there is no effective means to completely prevent or treat delirium. We suggest that the pathophysiological mechanisms of delirium include neuroinflammatory pathways, neurotransmitter imbalances, abnormal brain function under neuropathological conditions, neuroendocrine disruption, oxidative stress cascade, and intestinal microbiome dysregulation. These six mechanisms interact and intertwine with each other to promote the occurrence and development of ICU delirium.
